# Ecogenomics of Groundwater Phages Suggests Niche Differentiation Linked to Specific Environmental Tolerance

**DOI:** 10.1128/mSystems.00537-21

**Published:** 2021-06-29

**Authors:** Ankita Kothari, Simon Roux, Hanqiao Zhang, Anatori Prieto, Drishti Soneja, John-Marc Chandonia, Sarah Spencer, Xiaoqin Wu, Sara Altenburg, Matthew W. Fields, Adam M. Deutschbauer, Adam P. Arkin, Eric J. Alm, Romy Chakraborty, Aindrila Mukhopadhyay

**Affiliations:** a Biological Systems and Engineering, Lawrence Berkeley National Laboratory, Berkeley, California, USA; b Department of Energy Joint Genome Institute, Lawrence Berkeley National Laboratory, Berkeley, California, USA; c Environmental Genomics and Systems Biology Division, Lawrence Berkeley National Laboratory, Berkeley, California, USA; d Earth and Environmental Sciences, Lawrence Berkeley National Laboratory, Berkeley, California, USA; e Department of Plant and Microbial Biology, University of California, Berkeley, California, USA; f Energy Biosciences Institute, Berkeley, California, USA; g Department of Bioengineering, University of California, Berkeley, California, USA; h Department of Civil and Environmental Engineering, Massachusetts Institute of Technology, Cambridge, Massachusetts, USA; i Department of Biological Engineering, Massachusetts Institute of Technology, Cambridge, Massachusetts, USA; j Broad Institute of MIT Cambridge, Cambridge, Massachusetts, USA; k Center for Microbiome Informatics and Therapeutics, Massachusetts Institute of Technology, Cambridge, Massachusetts, USA; l Center for Biofilm Engineering, Montana State University, Bozeman, Montana, USA; m Department of Microbiology & Immunology, Montana State University, Bozeman, Montana, USA; Princeton University

**Keywords:** groundwater, virus, phage, plasmidome, viral sequences, metal resistance, antibiotic resistance, extrachromosomal DNA, viral genome, viral host

## Abstract

Viruses are ubiquitous microbiome components, shaping ecosystems via strain-specific predation, horizontal gene transfer and redistribution of nutrients through host lysis. Viral impacts are important in groundwater ecosystems, where microbes drive many nutrient fluxes and metabolic processes; however, little is known about the diversity of viruses in these environments. We analyzed four groundwater plasmidomes (the entire plasmid content of an environment) and identified 200 viral sequences, which clustered into 41 genus-level viral clusters (approximately equivalent to viral genera) including 9 known and 32 putative new genera. We used publicly available bacterial whole-genome sequences (WGS) and WGS from 261 bacterial isolates from this groundwater environment to identify potential viral hosts. We linked 76 of the 200 viral sequences to a range of bacterial phyla, the majority associated with *Proteobacteria*, followed by *Firmicutes*, *Bacteroidetes*, and *Actinobacteria*. The publicly available WGS enabled mapping bacterial hosts to several viral sequences. The WGS of groundwater isolates increased the depth of host prediction by allowing host identification at the strain level. The latter included 4 viruses that were almost entirely (>99% query coverage, >99% identity) identified as integrated in the genomes of Pseudomonas, *Acidovorax*, and *Castellaniella* strains, resulting in high-confidence host assignments. Lastly, 21 of these viruses carried putative auxiliary metabolite genes for metal and antibiotic resistance, which might drive their infection cycles and/or provide selective advantage to infected hosts. Exploring the groundwater virome provides a necessary foundation for integration of viruses into ecosystem models where they are key players in microbial adaption to environmental stress.

**IMPORTANCE** To our knowledge, this is the first study to identify the bacteriophage distribution in a groundwater ecosystem shedding light on their prevalence and distribution across metal-contaminated and background sites. Our study is uniquely based on selective sequencing of solely the extrachromosomal elements of a microbiome followed by analysis for viral signatures, thus establishing a more focused approach for phage identifications. Using this method, we detected several novel phage genera along with those previously established. Our approach of using the whole-genome sequences of hundreds of bacterial isolates from the same site enabled us to make host assignments with high confidence, several at strain levels. Certain phage genes suggest that they provide an environment-specific selective advantage to their bacterial hosts. Our study lays the foundation for future research on directed phage isolations using specific bacterial host strains to further characterize groundwater phages, their life cycles, and their effects on groundwater microbiome and biogeochemistry.

## INTRODUCTION

Viruses are known to influence the structure and diversity of microbial communities by infection and lysis of microbial cells. Their influence has been widely studied in aquatic communities (1), where they are predicted to infect approximately one-third of seawater microbes at any given time (2). In marine ecosystems, major biogeochemical cycles are known to be influenced by viruses affecting community composition, metabolic activity, and evolutionary trajectories (2, 3). As the recent focus on exploration of viruses in aquatic environments has been on marine ecosystems (4–9), freshwater environments remained mostly unexplored despite their importance as a drinking water supply (10). Most studies of viral diversity in freshwater systems have been conducted in lakes, across all continents and from pole to pole (11–15). Collectively, these studies revealed a large diversity of viruses specific to and/or mostly identified in freshwater ecosystems, mostly phages with double-stranded DNA (dsDNA) genomes, but also including eukaryotic viruses and phages with single-stranded DNA (ssDNA) and RNA genomes (16–18). Comparatively, viral diversity in groundwater systems has been much less studied, but recent metagenomic studies suggested that groundwater viral communities were clearly distinct from other freshwater environments, that their diversity and structure reflected changes in environmental parameters, including especially pH level and the presence of contaminants, and that viruses may significantly influence groundwater microbe dynamics (19, 20). The Oak Ridge Field Research Center (ORFRC) (21–23) is a well-studied U.S. Department of Energy site that includes groundwater areas with and without metal contamination, referred to as the contaminated and background sites, respectively. It has been well characterized in terms of the physical parameters, microbiome distribution, and fluctuation in response to different environmental stresses and thus served as an excellent model groundwater system for studies. We chose this environment to study the incidence of viruses in groundwater microbiome.

Identification of viral sequences in the environment is difficult given the lack of approaches similar to rRNA gene profiling in bacteria and their isolation remains challenging because of the difficulties in identifying the bacterial host(s) and our limited ability to culture them. Recently, research has been directed toward exploring viral diversity from metagenome data (7, 24, 25), thus circumventing these limitations and providing direct insights into the composition of environmental viral communities (26). In this study, we explored an alternate method to sifting through large amounts of chromosomal DNA sequences to find viral sequences by specifically searching circular DNA sequence data generated from the plasmidome analysis. Specifically, we mined the plasmidome data from a well-characterized groundwater system and analyzed the resulting viral sequences complete with genomic and ecological contexts.

## RESULTS AND DISCUSSION

### New viruses detected in the circular DNA data sets.

**FIG 1:**
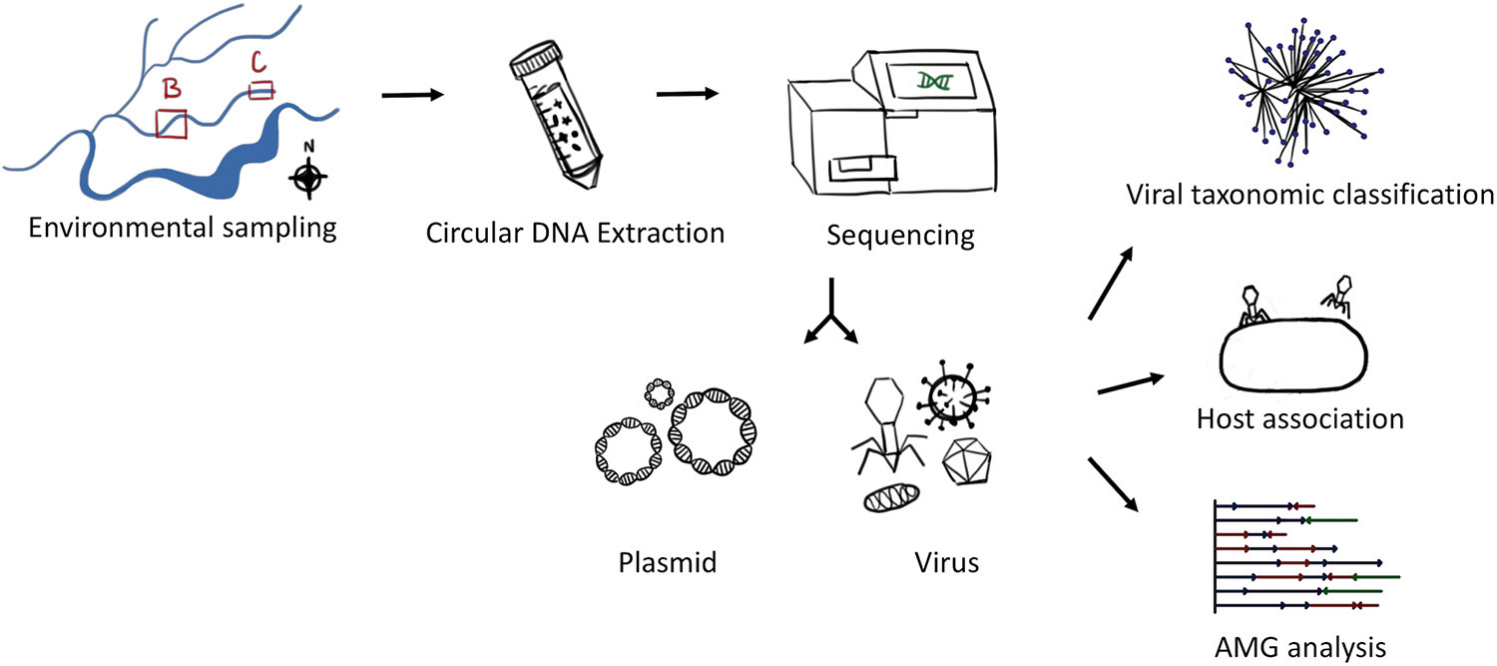
Overview of the study. Groundwater from the Oak Ridge Field research site from background (B) and contaminated (C) areas was filtered and subjected to circular DNA extraction. Sequencing, assembly, and annotation resulted in identification of both plasmids and viral genomes. The viral genomes were subjected to viral cluster analysis to study the virus types, host association analysis to get a prediction of bacteria they might infect, and auxiliary metabolite analysis (AMG) to study what functional genes they carry.

**FIG 2:**
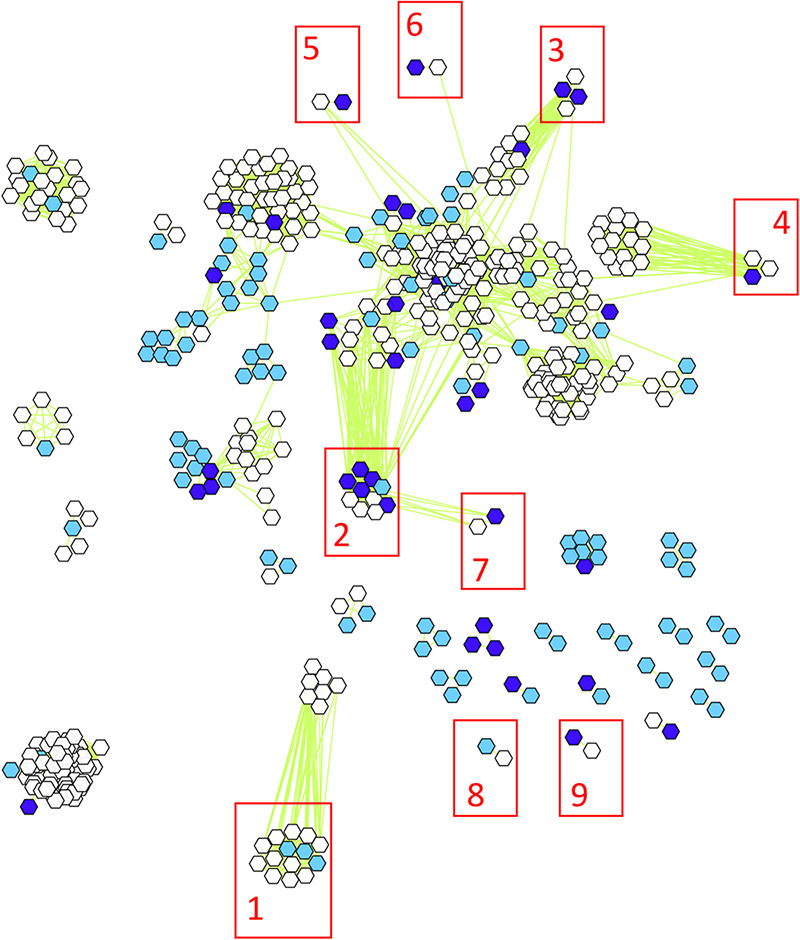
vConTACT-generated viral cluster map depicting clustering of 85 viral sequences from background (light blue) and contaminated (dark blue) groundwater, along with known virus reference genomes (white). The 9 viral clusters that contain known viruses are annotated on the figure as follows: 1, *Microviridae*; 2, *Podoviridae* (*Caudovirales*); 3, *Myoviridae* (*Caudovirales*); 4, *Myoviridae* (*Caudovirales*); 5, *Podoviridae* (*Caudovirales*); 6, *Siphoviridae* (*Caudovirales*); 7, *Podoviridae* (*Caudovirales*); 8, *Inoviridae*; 9, *Myoviridae* (*Caudovirales*). The green lines show vContact pairwise similarity scores. The order and distance between different viruses are arbitrarily selected values.

**FIG 3:**
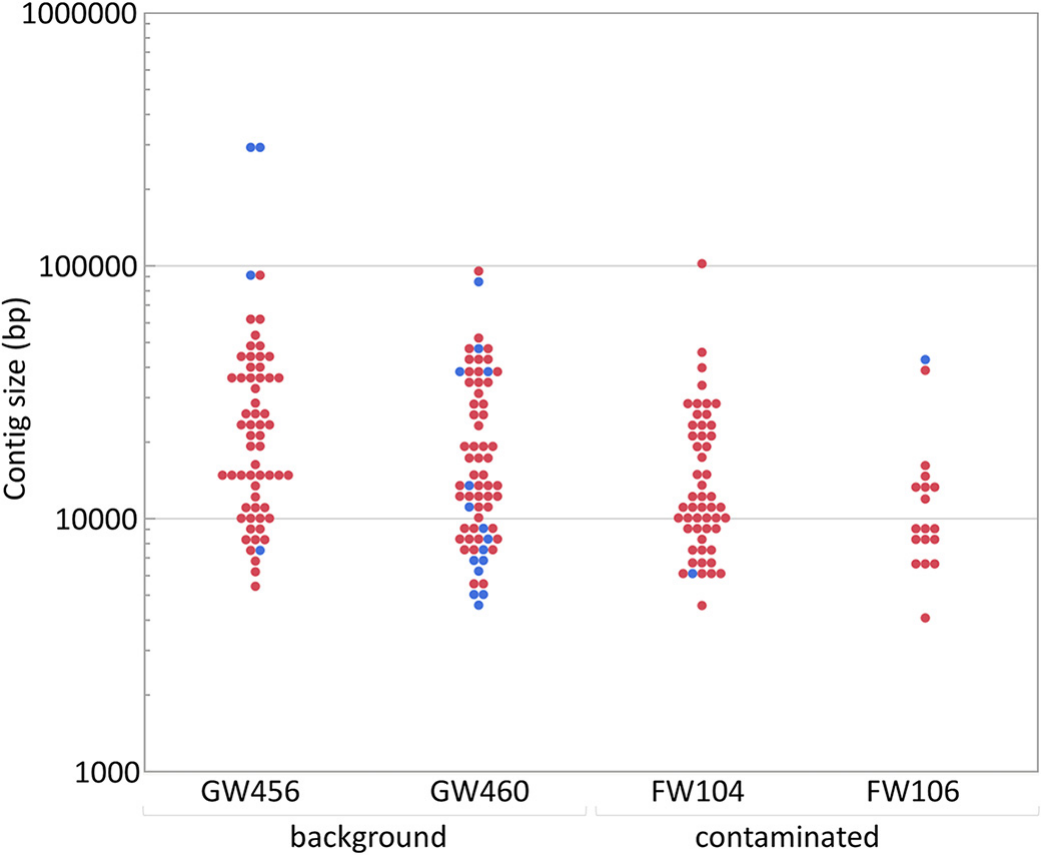
Size distribution of viruses from the background and contaminated groundwaters. The circular viral sequences are depicted in blue, while the rest are in red.

To study groundwater viruses, we leveraged existing data focused on extrachromosomal circular DNA templates by identifying viruses from plasmidome data sets (Fig. 1). This method primarily identifies active (intracellular) and lysogenic phages. Viruses and plasmids can coexist stably, support the transfer of each other to new hosts (27), or even form a hybrid (28). Given that both can be found as extrachromosomal circular DNA molecules, we used VirSorter, a tool designed to predict bacterial and archaeal virus sequences on the plasmidome assemblies (29), and identified 200 sequences as groundwater viral sequences from 13,770 plasmidome contigs (Fig. S1A). We then categorized viral sequences into viral clusters (approximately equivalent to known viral genera) using shared gene content information and network analytics (30, 31). Clustering of the 200 groundwater viral sequences with publicly available bacterial and archaeal viruses revealed that 85 groundwater viral genomes formed 41 viral clusters with at least one representative of groundwater virus (Table S3). Of these 41 clusters, 9 included a reference viral genome (Fig. 2) and 32 were putative new viral genera. The details on the size of different clusters are depicted in Fig. S1B. The largest identified virus was a circular 296,356-bp contig (see virus size distribution in Fig. 3) and was part of a novel viral cluster. Although more viral sequences were identified from the background than contaminated groundwaters, the fractions of all contigs identified as viral sequence were similar across both sites (Fig. S1A). There was little overlap between viral clusters from background and contaminated sites, with only 3 instances of viral sequences being in the same cluster from contaminated and background sites. Thus, the 200 groundwater viruses spanned a wide variety of sizes and included representatives of both known and novel viral genera.

10.1128/mSystems.00537-21.1 FIG S1 (A) Breakdown of the all the contigs tested, with the those predicted to be viral highlighted in blue. (B) Distribution of viral clusters and the number of phages in each cluster. Download FIG S1, TIFF file, 8.2 MB. Copyright © 2021 Kothari et al. 2021 Kothari et al. https://creativecommons.org/licenses/by/4.0/ This content is distributed under the terms of the Creative Commons Attribution 4.0 International license.

10.1128/mSystems.00537-21.6 TABLE S3 Information on all 41 viral clusters that groundwater viruses fall into. This includes 32 novel clusters and 9 known clusters including known phages. Download Table S3, XLSX file, 0.04 MB. Copyright © 2021 Kothari et al. 2021 Kothari et al. https://creativecommons.org/licenses/by/4.0/ This content is distributed under the terms of the Creative Commons Attribution 4.0 International license.

Certain aspects of the viral clusters provide evidence of optimal clustering of groundwater viruses. All 9 viral clusters with known reference viral genomes were circular DNA viruses. The viral cluster with 14 representatives had 11 representatives belonging to the family *Microviridae*, subfamily *Gokushovirinae*, which are 4.5- to 6-kb, circular single-stranded DNA viruses. Interestingly, the 3 viral contigs that are clustered are from the background site and are also in the same size range (4.61, 4.78, and 5.09 kb). At least one virus (GW460_nc_scaffold_3616, 8,250 bp) from the background site is an inovirus (5 to 15 kb, circular single-stranded DNA genomes with rod-shaped or filamentous virions [32]) clustering with known inovirus *Ralstonia* phage 1 NP-2014. The genomes of inoviruses are known to be chromosomally integrated or replicated as a plasmid (33), which may be why this virus was recovered from plasmidome data.

### Host predictions.

**FIG 4:**
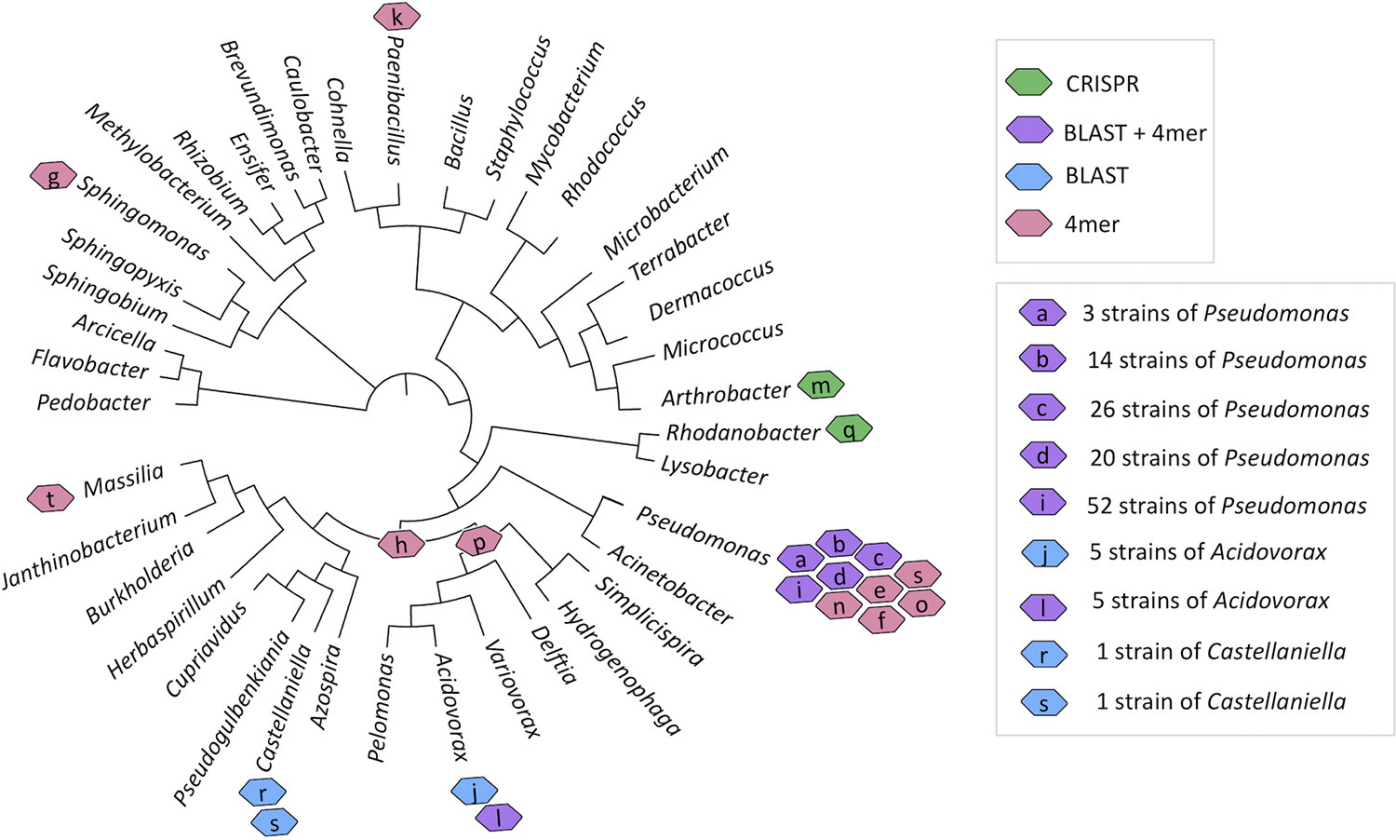
Viral host predictions based on BLAST, high-stringency BLAST (BLAST99), tetranucleotide frequency (4-mer), and CRISPR methods using whole-genome sequence (WGS) information from 261 ORFRC bacterial isolates. The details of the 20 viruses (“a” to “t”) are provided in Table S4. The viruses “h” and “p” are assigned to hosts in the class *Betaproteobacteria* and the family *Comamonadaceae*. The rest of the viruses are assigned to the indicated genera. The phylogenetic tree was made from 16S rRNA sequence of 261 ORFRC strains. The viral sequence “s” appears twice because it was predicted to infect two different genera based on the different prediction methods.

Once we identified viral genomes and their clusters, we sought to identify the range of hosts that these viruses infect. Using the 261 ORFRC bacterial isolates, we were able to assign bacterial hosts to 20 viral genomes (Fig. 4) of the 200, indicating that we were able to predict hosts for 10% of the viral genomes identified (Table S4). As expected, the maximum number of predictions were made using tetranucleotide frequency (16 bacterial strains predicted as hosts), followed by BLAST (9 bacterial strains predicted as hosts) and CRISPR (2 bacterial strains predicted as hosts) analysis (Fig. S2A). All 9 viral sequences that had a bacterial host genus predicted via BLAST also had strain-level predictions using BLAST99. An example of host prediction via BLAST99 is depicted in Fig. S2B, where the entire viral sequence was found in five different *Acidovorax* strains. Interestingly, 7 viral genomes were assigned hosts using both BLAST and tetranucleotide frequency methods, and 6 of them were predicted to have the same bacterial-host genus, increasing the confidence in their host prediction. Out of 20, 10 viral genomes had Pseudomonas predicted as the bacterial host, and overall, 18 viral genomes were assigned to *Proteobacteria*. This could be attributed to the fact that of 261 ORFRC isolates, over 50% were pseudomonads and over 85% were *Proteobacteria*, making it easier to identify them as host strains. Thus, several ORFRC bacterial genus and strains belonging to the phyla *Proteobacteria*, *Actinobacteria*, and *Firmicutes* were predicted as hosts for the viruses.

10.1128/mSystems.00537-21.2 FIG S2 (A) Details of numbers of viral sequences for which hosts are predicted using various bacterial-host prediction methods on ORFRC and NCBI strains with whole-genome sequences. The Venn diagram shows the overlap of host prediction for viral sequences when the ORFRC and NCBI strains are used. (B) Visual depiction of a single viral sequence being entirely encoded in multiple bacterial strains. About 99.9% of the viral sequence GW456_nc_scaffold_4557 (E value = 0) could be found in 5 *Acidovorax* strains isolated from ORFRC. Download FIG S2, TIFF file, 9.7 MB. Copyright © 2021 Kothari et al. 2021 Kothari et al. https://creativecommons.org/licenses/by/4.0/ This content is distributed under the terms of the Creative Commons Attribution 4.0 International license.

10.1128/mSystems.00537-21.7 TABLE S4 Host associations. Predictions based on ORFRC groundwater bacterial whole-genome sequences and predictions based on NCBI bacterial whole-genome sequences. Breakdown of host assignments made using NCBI and ORFRC bacterial whole-genome sequences and comparison of host prediction between members of the same viral cluster using NCBI and ORFRC bacterial whole-genome sequences. Download Table S4, XLSX file, 0.5 MB. Copyright © 2021 Kothari et al. 2021 Kothari et al. https://creativecommons.org/licenses/by/4.0/ This content is distributed under the terms of the Creative Commons Attribution 4.0 International license.

We also leveraged the complete archaeal and bacterial genome sequences available on NCBI, to make predictions of bacterial hosts for the 200 viral genomes. No hits were found using the 311 archaeal strains. Using the 14,028 bacterial strains, host predictions could be made for about 36.5% (73 of 200) of the viral genomes, with a vast majority assigned to the phylum *Proteobacteria* (Table S4). Other bacterial hosts were in the phyla *Actinobacteria*, *Bacteroidetes*, *Firmicutes*, *Chlamydiae*, and *Chloroflexi*. Again, the maximum number of predictions (71) were made using tetranucleotide frequency, followed by BLAST analysis (5 bacterial strains predicted as hosts) (Fig. S2A). The BLAST99 analysis had no hits, so strain-specific bacterial host predictions were not made. Interestingly, all 5 viral genomes that had predictions with BLAST also had predictions using the tetranucleotide frequency method. Although a higher number of viral sequences could be assigned to bacterial hosts using whole-genome sequences (WGS) from NCBI than ORFRC, the probability of finding a host for every bacterial WGS tested was higher with ORFRC strains (7.6%) than NCBI strains (0.5%), highlighting the benefits of including bacterial strains from the same environment as the viral sequence itself. More importantly, strain-specific host assignments could only be made using groundwater bacterial isolates, and such high-resolution host assignment is important when designing experiments aimed at isolating specific phages.

**FIG 5:**
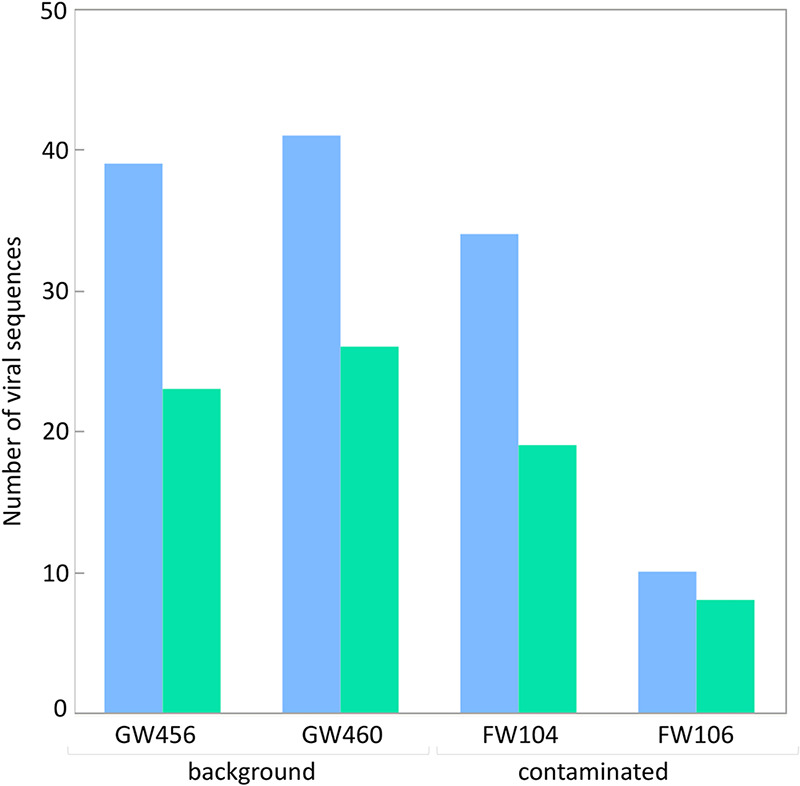
Compilation of viral sequences across the background and contaminated groundwater sites based on availability of bacterial host prediction (green indicates that the bacterial host was predicted, while blue indicates a lack of available bacterial-host prediction).

Using the ORFRC and NCBI strain host predictions together, we were able to assign bacterial hosts to 38% (76 of 200) of the viral genomes (Fig. 5). Around 17 viruses had host predictions based on both ORFRC and NCBI strains (Fig. S2A), with the same bacterial phyla predicted as hosts (Table S4). Differences like this could be attributed to the nonoverlapping nature of the strains from NCBI and ORFRC and differences in the strength of host prediction methods. Next, we compared host prediction between members of the same viral cluster (Table S4). The bacterial host predictions mostly remained consistent within the same viral cluster. The minor discrepancy seen in the viral clusters can likely be explained on further analysis; for instance, the exceptional viral cluster VC_138_0 consists of 10 members, with six being groundwater viruses, and their hosts were predicted to be either *Burkholderiales* or *Pseudomonadales* based on the prediction method used. Interestingly, the four known viruses they cluster with were *Bordetella* virus BPP1, Pseudomonas phage AF, Pseudomonas phage vB_PaeP_Tr60_Ab31, and Xanthomonas citri phage CP2, indicating that members of this cluster infect both *Burkholderiales* and *Pseudomonadales*. Thus, consistent patterns of host prediction emerge within the same viral cluster.

### Presence of metabolic genes.

**FIG 6:**
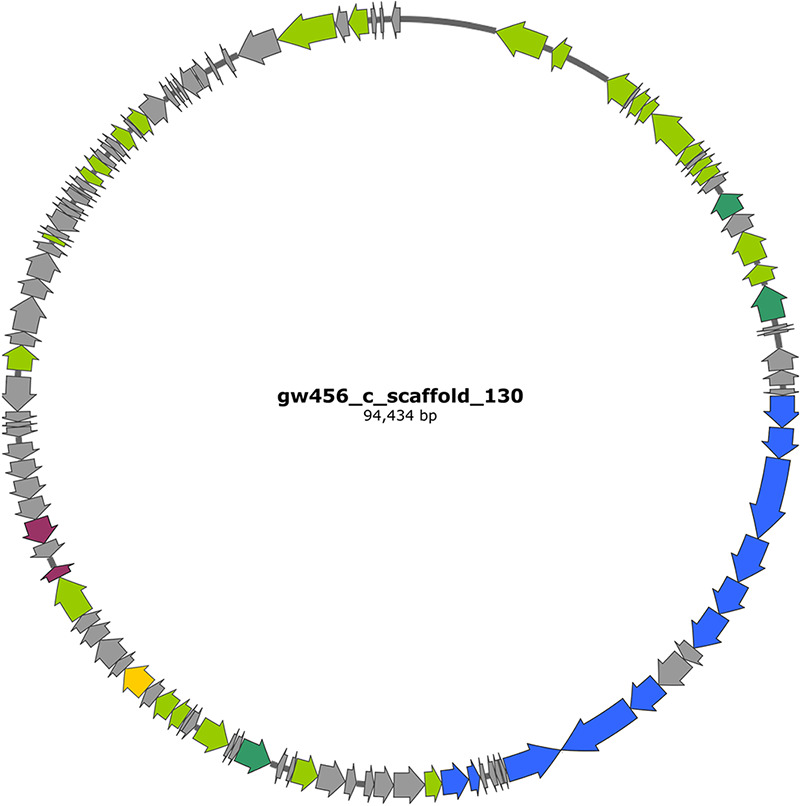
Example of a viral contig carrying auxiliary metabolite genes. Map of the virus (gw456_c_scaffold_130) from background groundwater with phage-related genes highlighted in green (darker green represents true hallmark genes of viruses), metal (copper, cobalt, zinc, cadmium, lead, mercury, and arsenic) resistance genes highlighted blue, antibiotic (spectinomycin and fosfomycin) resistance genes highlighted in pink, and the metabolism (lactate dehydrogenase) gene in yellow. The viral contig was annotated via Prokka in KBase and the annotations for virus-associated genes were updated on the map using VirSorter predictions; details are in Table S6.

In addition to affecting groundwater biogeochemistry through their physical contribution to dissolved organic matter and the lysis of their hosts, viruses can also affect the diversity and function of microbial populations through the incorporation and expression of auxiliary metabolic genes (AMGs) (4, 34). AMG definitions are still being refined (35), but generally, these genes are not involved in viral replication or structure but instead allow viruses to directly manipulate host metabolism during infection. Examination of all the viral sequences revealed a total of 1,486 hits classified into known Pfam categories (Fig. S3). Exploring Pfam domains associated with microbial metabolism resulted in the identification of 51 unique putative AMGs (Table S5). Since these viral sequences are from a site where metal and antibiotic resistance genes are routinely seen (36–38), all the unique PFAM hits were manually curated to identify metal and antibiotic resistance genes (Table S5). We found that the metal resistance genes identified as putative AMGs were those providing resistance to copper, while the antibiotic resistance genes in the list of putative AMGs were annotated as multiresistance beta-lactamase, providing resistance to β-lactam antibiotics; multidrug efflux pumps in the AcrB/AcrD/AcrF family, providing multidrug resistance; and streptomycin adenylyltransferase, providing resistance to streptomycin. An excellent example is viral sequence GW456_c_scaffold_130, which was annotated to encode metal and antibiotic resistance genes along with signature phage genes, consistent with a complete phage genome (Fig. 6; annotation details are provided in Table S6). The compilation of all the data discussed is available in Table S7. To the best of our knowledge, this is the first report of the presence of metal and antibiotic resistance genes on viral sequences. The presence of metal and antibiotic resistance genes suggests that groundwater viruses may manipulate metal tolerance mechanisms, enabling their hosts to adapt to environmental stressors.

10.1128/mSystems.00537-21.3 FIG S3 Major categories of 1,486 virus genes predicted via Pfam database. The hits were sorted into categories based on gene names as previously established based on AMGs seen in ocean viruses (57). FIG S3, PDF file, 0.2 MB Copyright © 2021 Kothari et al. 2021 Kothari et al. https://creativecommons.org/licenses/by/4.0/ This content is distributed under the terms of the Creative Commons Attribution 4.0 International license.

10.1128/mSystems.00537-21.8 TABLE S5 Details of all the Pfam domains detected on the 200 viral sequences and Pfam domains related to metal resistance-, antibiotic resistance-, and toxin-antitoxin-encoding genes. Download Table S5, XLSX file, 0.07 MB. Copyright © 2021 Kothari et al. 2021 Kothari et al. https://creativecommons.org/licenses/by/4.0/ This content is distributed under the terms of the Creative Commons Attribution 4.0 International license.

10.1128/mSystems.00537-21.9 TABLE S6 Details of the 121 genes on the viral sequence GW456_c_scaffold_130, including their location, size, directionality, and description. Download Table S6, XLSX file, 0.02 MB. Copyright © 2021 Kothari et al. 2021 Kothari et al. https://creativecommons.org/licenses/by/4.0/ This content is distributed under the terms of the Creative Commons Attribution 4.0 International license.

10.1128/mSystems.00537-21.10 TABLE S7 Details of all 200 viral sequences with compilation of all analyses, including virus size, viral cluster, host prediction, and AMG analysis. Download Table S7, XLSX file, 0.02 MB. Copyright © 2021 Kothari et al. 2021 Kothari et al. https://creativecommons.org/licenses/by/4.0/ This content is distributed under the terms of the Creative Commons Attribution 4.0 International license.

### Conclusion.

We demonstrate identification of novel viruses by leveraging plasmidome data for exploring environmental viral communities. Our analyses revealed the presence of novel viruses, likely representing new viral genera, in the underexplored groundwater environment. Using different data sets, we achieved bacterial host predictions for a substantial number of the viral sequences. Several of these phages carry genes related to signaling and tolerance mechanisms, thus likely augmenting ecosystem function by modifying the metabolism of their bacterial hosts. Interestingly, we found genes annotated to provide tolerance to metals, which is significant source of stress at this site. These predictions form the basis of future work on guiding phage isolation efforts and functional assessment of virus-host linkages. The ability to isolate phages would open new avenues for targeted manipulation of specific subsets of bacteria, thus allowing the systematic dissection of a microbiome for probing community dynamics and function.

## MATERIALS AND METHODS

### Groundwater sample collection and sequencing analysis.

The groundwater samples were obtained from the Oak Ridge Field Research Center (ORFRC) site (21–23) and included samples from metal-contaminated (wells FW104 and FW106) and background (wells GW456 and GW460) areas. The metal-contaminated site was characterized by chronically high concentrations of radionuclides (e.g., uranium), nitric acid, organics, salts, mercury, and other heavy metals (36). The level of contaminants in the background site is available in the supplemental material of a previous study (37) exploring the plasmidome. The plasmidome study (37) described the circular DNA isolation (plasmidome analysis) procedure from 5 liters of groundwater from background sites (GW456 and GW460), followed by sequencing, assembly, annotation, and other analyses. Additionally, for the present study, we also used plasmidome sequence data from two contaminated-site samples comprising 8 liters groundwater from FW104 and FW106 and subjected to the same analysis (unpublished data; sequencing data available via MG-RAST IDs mgm4830571.3 and mgm4830867.3).

To extract DNA from bacteria on a filter, we used a modified version of the alkaline hydrolysis plasmid DNA isolation method (39). The remnant linear DNA fragments were removed by plasmid-safe ATP-dependent DNase (Epicentre Biotechnologies, Madison, WI) at 37°C for 48 h with double the recommended ATP and enzyme amounts. The lack of chromosomal DNA contamination was confirmed by PCR with degenerate 16S rRNA primers. The plasmid DNA was amplified with phi29 DNA polymerase (New England Biolabs, Ipswich, MA) as previously described (40) for 6 days at 18°C. This was followed by ethanol precipitation and use of a NanoDrop instrument to concentrate and quantify the DNA. The lack of chromosomal DNA contamination in plasmid DNA extracted from groundwater samples F and G was confirmed by PCR with degenerate 16S rRNA primers.

All DNA was sequenced using Illumina MiSeq reagent v3 kit (paired-end protocol). Trimmomatic 0.36 (41) (http://www.usadellab.org/cms/?page=trimmomatic) was used to trim the reads with the following parameters: IlluminaClip:TruSeq3-PE.fa:2:30:10, Leading:3 Trailing:3 SlidingWindow:4:15 MinLen:36. IDBA-UD (42) (used for *de novo* read assembly with the parameter “–pre_correction”). Assembled sequences were searched against the SILVA 16S rRNA database (43) using BLASTN; all scaffolds with >200-bp identity to 16S rRNA were removed from further analysis to reduce any chromosomal DNA contamination. This step significantly reduces chromosomal contamination, but given the nature of the study, is not possible to eliminate all chromosomal-DNA contamination in the data set. We modified a pipeline method for postassembly detection of circularity among scaffolds with the following criteria to identify the complete closed circular scaffolds referred to as “circular_scaffolds” or simply circular plasmids: (i) scaffold length of >2 kb, (ii) >34-bp homology (E value > 1e−5) at the ends of the scaffold in the correct direction, and (iii) at least two read pairs mapping on opposite ends of the contig, a maximum of 500 bp from the end.

### Identification of viral contigs.

After sequencing, the assembly of all contigs (including plasmid and viral DNA), along with prediction of circular sequences using bioinformatic analyses, was performed as described previously (37). Briefly, all plasmid sequences obtained were subjected to a pipeline method for postassembly detection of circularity among scaffolds, and any scaffolds failing this are termed noncircular contigs, to distinguish them from plasmid sequences which met the criteria. All circular contigs along with noncircular contigs encoding more than 10 proteins were subjected to VirSorter analysis (29), an iVirus tool available via CyVerse (44) for identification of viruses. VirSorter was used to identify and remove microbial contigs using the “virome decontamination” mode, with every contig that was not identified as viral being considered a microbial contig. The final set of viral contigs was formed by compiling sequences detected as VirSorter categories 1 and 2 along with prophage categories 4 and 5 (Table S1). Thus, we focused on the 200 viral sequences with high-confidence assignments (VirSorter categories 1, 2, 4, and 5) and ignored the low-confidence assignments (VirSorter categories 3 and 6). VConTACT2 (45) was used to perform viral cluster analysis, and the results were visualized using Cytoscape (46). Since the groundwater from background site was spiked with Desulfovibrio vulgaris Hildenborough (ATCC 29579), Escherichia coli DH1 (ATCC 33849), and E. coli strain J-2561 as controls for the plasmidome study, any viruses associated with these strains were removed from the analysis. Given that the DNA isolation procedure concentrated on targeted isolation of circular DNA, there is an expected inherent bias in identifying circular dsDNA viral sequences from this data set. All viral genomes are available at https://kbase.us/n/76973/17/.

10.1128/mSystems.00537-21.4 TABLE S1 Information on all contigs identified as viruses. These are sorted into categories phage (1 to 3) and prophage (4 to 6). Only the higher-confidence categories 1, 2, 4, and 5 are considered phage in this study. Download Table S1, XLSX file, 0.07 MB. Copyright © 2021 Kothari et al. 2021 Kothari et al. https://creativecommons.org/licenses/by/4.0/ This content is distributed under the terms of the Creative Commons Attribution 4.0 International license.

### Generation of host database. (i) Generation of host database from ORFRC bacterial isolates.

*(a) Isolation of bacterial strains.* The bacterial isolates were obtained via direct-plating under aerobic or anaerobic conditions at 25 to 30°C in the dark, using ORFRC groundwater or sediment extract as the inoculum, or via two-step isolation: enrichment incubation of 1 ml groundwater in 9 ml liquid medium aerobically for 2 weeks followed by direct plating for isolation. A subset of isolates were obtained from biofilm reactors (CDC reactors) that were fed ORFRC groundwater and had nonporous glass beads (30 μm) as a matrix for biofilms in coupons. Water or beads from the reactors were used as the inoculum. For direct plating, rich-medium (Luria-Bertani, tryptic soy; R2A; Eugon, Winogradsky) agar plates or basal-medium (4.67 mM ammonium chloride, 30 mM sodium phosphate, with vitamin and mineral mixes as previously described [47]) agar plates were used. The liquid medium for enrichment incubation was filtered groundwater amended with one or a combination of the following carbon sources: glucose (5 mM), acetate (5 mM), benzoate (0.5 mM), Casamino Acids (10 μg/ml), bacterial cell lysate, and sediment-extracted dissolved organic matter. After direct plating, single colonies were picked and regrown in liquid medium for 16 to 48 h until the culture reached mid-log phase. Then a portion of the culture was used to extract DNA for 16S rRNA-based identification, and the rest was cryopreserved with sterile glycerol (to a final concentration of 30%), flash frozen with liquid nitrogen, and stored at −80°C.

*(b) Whole genome sequencing and de novo assembly.* Cultures were revived from glycerol stocks by streaking onto Luria-Bertani or R2A agar plates. Individual colonies developed at 30°C over 48 h and were then inoculated into corresponding liquid media and grown at 30°C for 48 h. The cultures were centrifuged, and the genomic DNA was extracted using the Qiagen DNeasy kit (Qiagen, Venlo, the Netherlands) according to the manufacturer’s instructions. All samples were eluted in Qiagen’s AE buffer: 10 mM Tris-Cl, 0.5 mM EDTA (pH 9.0). Genomic DNA was stored at −20°C followed by transfer into a 384-well plate for automated library preparation. The isolated genomic DNA was normalized to 0.2 ng/μl in 10 mM Tris (pH 8.0), and libraries were prepared using the Illumina Nextera XT kit at 1/12 reaction volume on a SPT Labtech Mosquito HV. Final libraries were purified using solid-phase reversible immobilization beads, and sequenced on an Illumina NextSeq 500 with 150-bp paired-end reads. The program Cutadapt v1.12 was used to remove adapter sequences with the parameters -a CTGTCTCTTAT -A CTGTCTCTTAT (48). We performed sliding-window quality filtering with Trimmomatic v0.36 (parameters: -phred33 LEADING:3 TRAILING:3 SLIDINGWINDOW:5:20 MINLEN:50) (41). All genomes were assembled *de novo* using SPAdes v3.9.0 with the following options (-k 21,33,55,77 –careful) (49). Genome quality was validated with the program checkM v1.0.6 using the lineage_wf pipeline with default parameters (47), and all draft genomes met the criteria of contamination of <10% and completeness of >95%. The 16S rRNA gene sequences were recovered with RNAmmer v1.2 (–S bac –m ssu) and taxonomically classified with SINTAX (usearch v9.2.64) against the Ribosomal Database Project (RDP) (50) 16S rRNA gene training set v16 with species names and the following parameters: –strand both –sintax_cutoff 0.8 (51, 52). The whole-genome sequences (WGS) of 261 bacterial isolates (details in Table S2) from ORFRC were combined to form a database for further bioinformatic analyses. The WGS of the 261 strains are available at https://kbase.us/n/63776/35 with the DOI 10.25982/63776.53/1637360.

10.1128/mSystems.00537-21.5 TABLE S2 Details of the 261 bacterial strains isolated from ORFRC. Download Table S2, XLSX file, 0.08 MB. Copyright © 2021 Kothari et al. 2021 Kothari et al. https://creativecommons.org/licenses/by/4.0/ This content is distributed under the terms of the Creative Commons Attribution 4.0 International license.

### (ii) Generation of host database from NCBI bacterial and archaeal isolates.

A genome database of putative hosts for the viruses was generated including all archaeal (311 assembled complete genomes, downloaded in September 2019) and bacterial (14,028 assembled complete genomes, downloaded in August 2019) genomes from NCBI Assembly. The taxonomic affiliation of the genomes was taken from the NCBI taxonomy.

### Host prediction and diversity.

Three different previously published approaches (53, 54) for predicting hosts based on examining similarities between (i) a bacterial genome-encoded CRISPR spacer and viral genome (55), (ii) viral and microbial genomes due to integrated prophages or gene transfers (56), and (iii) viral and host genome nucleotide signatures (here, tetranucleotide frequency similarity) (30) were used as described below. The confidence in assignment via these three methods to different clades in bacterial classification was estimated previously (57), with CRISPR-based predictions being the most accurate, while the tetranucleotide frequency-based predictions were the least accurate at the genus level.

### (i) BLAST-based identification of sequence similarity between viral contigs and host genome.

All 200 viral contigs were compared to all archaeal and bacterial genomes with BLASTn (threshold of 50 for bit score and 0.001 for E value), to identify regions of similarity between a viral contig and a microbial genome, indicative of a prophage integration or horizontal gene transfer. As previously established (54), host prediction was made when an NCBI genome displayed a region similar to the viral contig of ≥4.9 kb at ≥70% identity. When one viral sequence had hits to multiple bacterial strains, the top 5 hits (based on bit score) were analyzed to determine the last common ancestor clade. This clade was then assigned as the host to the virus. Based on this method, genus-level bacterial-host predictions were made. Bacterial strain-specific host predictions were made only when the entire virus was found to be present in the bacterial whole-genome sequence. In this case, BLAST with highly stringent parameters, referred to as BLAST99 (>99% query coverage, E value = 0, and >99% identity), was performed to query for the presence of an entire viral sequence in the host.

### (ii) Matches between viral contigs and CRISPR spacers.

CRISPR arrays were predicted for all ORFRC microbial genomes with CRISPR Recognition Tool (CRT) (58) using default settings (repeat settings used 3 minimum repeats, a minimum repeat length of 19, a maximum repeat length of 38, and a search window of 8, along with spacer settings using a minimum spacer length of 19 and a maximum spacer length of 48). We used previously published (54, 59) BLAST parameters for identifying the target of CRISPR spacers (i.e., using the BLASTn-short task, a maximum expect value of 1, a gap opening penalty of 10, a gap extension penalty of 2, a word size of 7, and dust filtering turned off). Given that the accuracy of this approach for detecting phage hosts strongly depends on the maximum number of mismatches allowed between the CRISPR spacer and the viral sequence, the results were filtered to allow 0 or 1 mismatch. Only the CRISPR spacers that matched viral sequences were then compared back with the bacterial WGS with no mismatch to come up with bacterial host predictions. Based on this method, strain-level bacterial-host predictions were made.

### (iii) Nucleotide composition similarity: comparison of tetranucleotide frequency.

Bacterial and archaeal viruses tend to have a genome composition close to the genome composition of their host, a signal that can be used to predict virus-host pairs (54, 57, 60). Here, canonical tetranucleotide frequencies (also referred to as 4-mers) were observed for all viral and host sequences using Jellyfish (61), and mean absolute error (that is, the average of absolute differences) between tetranucleotide-frequency vectors was computed with in-house Perl and Python scripts for each pair of viral and host sequence as previously reported (57). A viral contig was then assigned if the average of absolute differences (*d*) between tetranucleotide-frequency vectors was <0.001. When multiple strains had hits to one viral sequence, the top five hits (based on lowest distance) were analyzed to determine the lowest common ancestor of the group. This lowest common ancestor was then assigned as the host to the virus. Based on this method, genus-level bacterial-host predictions were made.

### Phylogenetic tree construction.

For constructing the phylogenetic tree using ORFRC isolates, the 16S rRNA sequences from all 261 strains were aligned using Muscle (62). The evolutionary history was inferred by using the maximum-likelihood method based on the Tamura-Nei model using MEGA7 (63). The tree with the highest log likelihood (−7,846.44) is shown. The initial tree(s) for the heuristic search was obtained automatically by applying Neighbor-Join and BioNJ algorithms to a matrix of pairwise distances estimated using the maximum composite likelihood (MCL) approach and then selecting the topology with superior log likelihood value. The analysis involved 255 nucleotide sequences. All positions containing gaps and missing data were eliminated. There were a total of 519 positions in the final data set. All branches were collapsed at the genus level. For the phylogenetic tree depicting NCBI isolates, existing trees were downloaded using NCBI Taxonomy and collapsed to genus levels.

### Viral sequence annotation.

A functional annotation of all virus-encoded predicted proteins was based on a comparison to the Pfam domain database v.32 (64) with HmmScan (65) (threshold of 30 for bit score and 10^−3^ for E value). The Pfam categories were assigned based on Pfam target name as previously described (57), and any Pfam target name not categorized earlier is referred to as “not categorized.” All contigs were also uploaded to KBase for annotation. To specifically identify metal and antibiotic resistance genes, all the unique Pfam target names and their descriptions were manually curated.
