# Supplementary figures and images for: Ecogenomics of Groundwater Phages Suggests Niche Differentiation Linked to Specific Environmental Tolerance
Source: mSystems. 2021 Jun 29;6(3):e00537-21. doi: 10.1128/mSystems.00537-21 (PMC8269241; doi:10.1128/mSystems.00537-21)

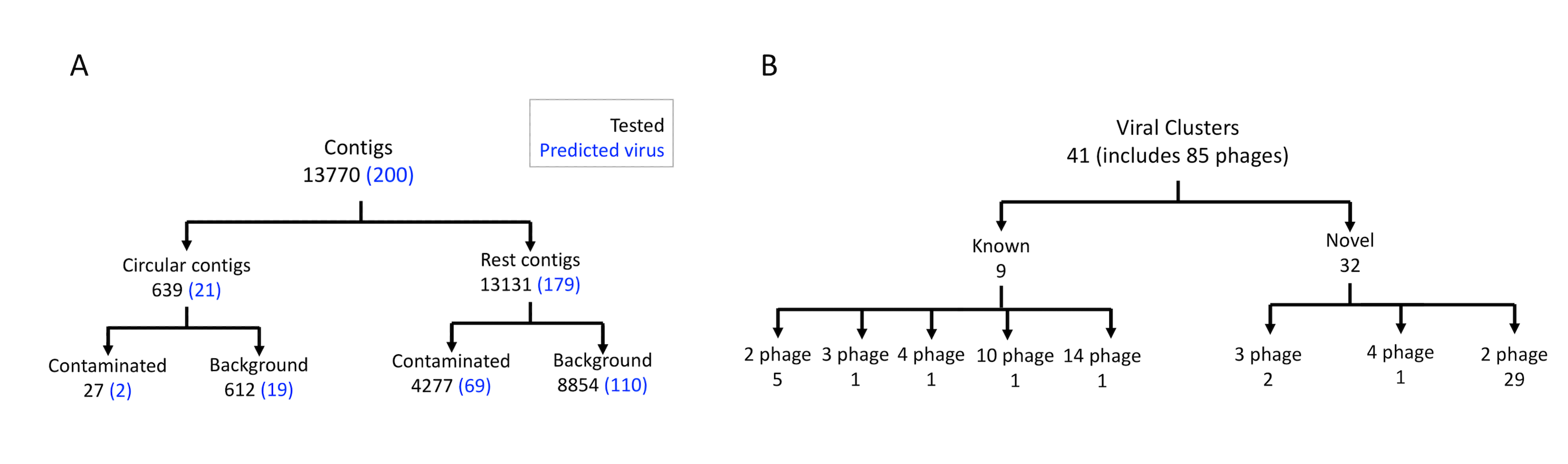

Supplement: FIG S1 [file msystems.00537-21-sf001.tif]

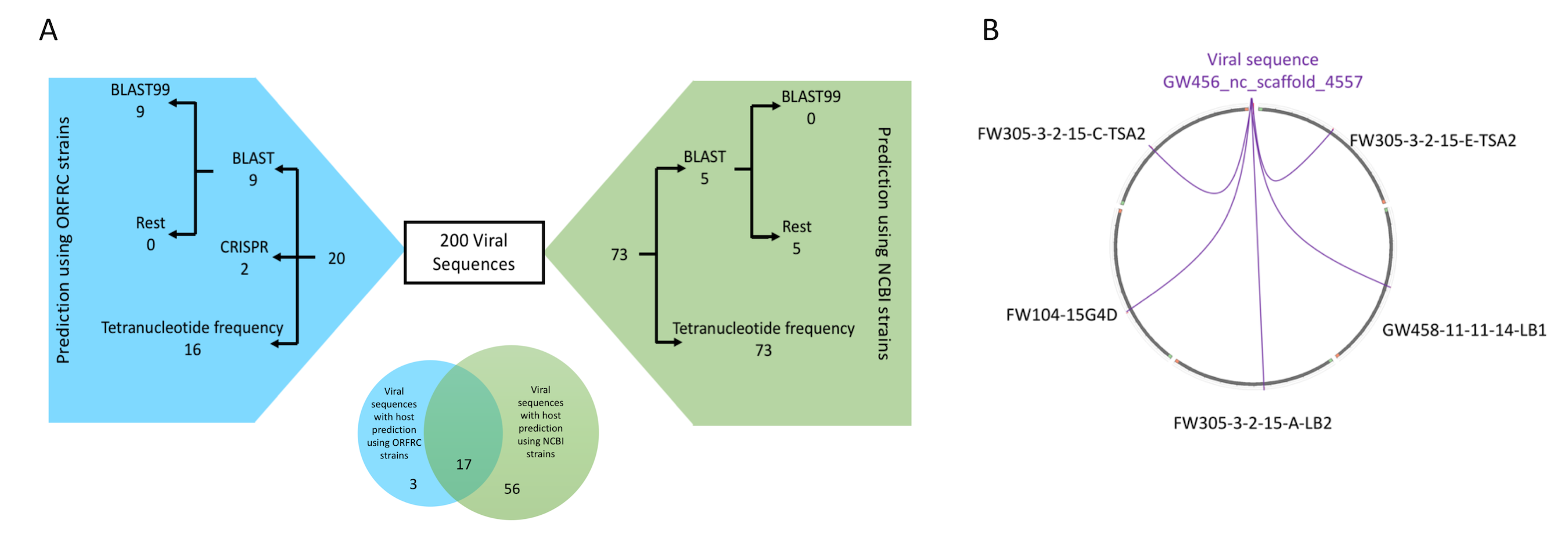

Supplement: FIG S2 [file msystems.00537-21-sf002.tif]
