# Supplementary material for: Ecogenomics of Groundwater Phages Suggests Niche Differentiation Linked to Specific Environmental Tolerance
Source: mSystems. 2021 Jun 29;6(3):e00537-21. doi: 10.1128/mSystems.00537-21 (PMC8269241; doi:10.1128/mSystems.00537-21)
Supplement: FIG S3 [file msystems.00537-21-sf003.pdf]

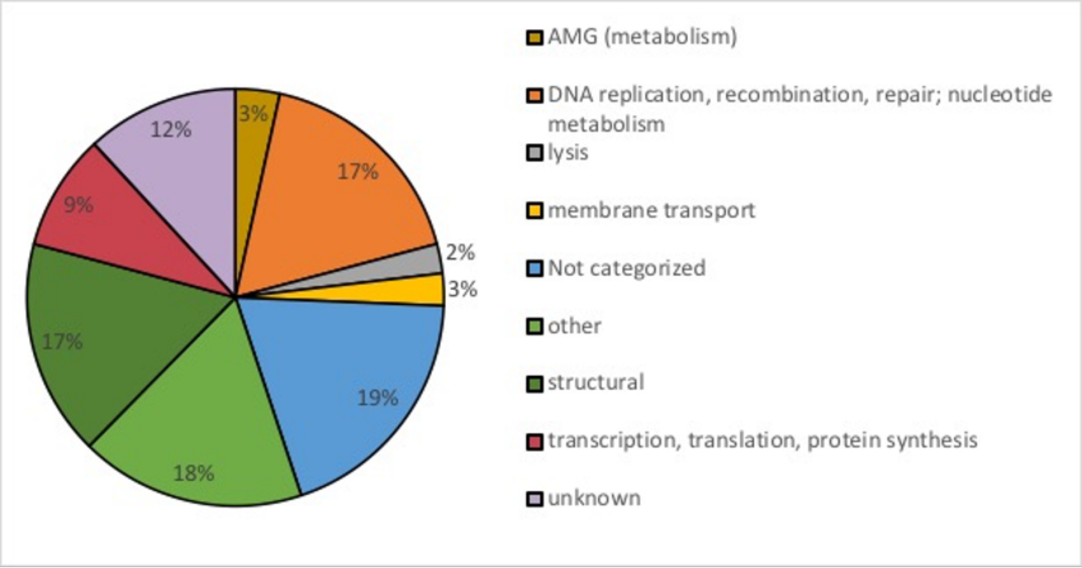

3%
17%
2%
3%
19%
18%
17%
9%
12%
AMG (metabolism)
DNA replication, recombination, repair; nucleotide metabolism
lysis
membrane transport
Not categorized
other
structural
transcription, translation, protein synthesis
unknown
